# Gum Arabic as a Cause of Occupational Allergy

**DOI:** 10.1155/2011/841508

**Published:** 2011-05-19

**Authors:** Arja Viinanen, Maija Salokannel, Kaija Lammintausta

**Affiliations:** ^1^Department of Pulmonary Diseases, Turku University Hospital, P.O. Box 52, 20521 Turku, Finland; ^2^Occupational Health Service Unit, Mehiläinen, 20520 Turku, Finland; ^3^Department of Dermatology, Turku University Hospital, P.O. Box 52, 20521 Turku, Finland

## Abstract

*Background*. Gum arabic is a potential sensitizer in food industry. *Methods*. We examined 11 candy factory workers referred to examinations due to respiratory and skin symptoms paying attention to exposure and sensitization to gum arabic. Skin tests, pulmonary function tests, and respiratory provocation tests were carried out as indicated by the symptoms and findings. *Results*. Occupational asthma, caused by gum arabic was diagnosed in 4/11 candy factory workers and two of them had also occupational contact urticaria and one had occupational rhinitis. One of them had oral symptoms associated with ingestion of products containing gum arabic. *Conclusions*. Airborne exposure to gum arabic may cause sensitization leading to allergic rhinitis, asthma, and urticaria.

## 1. Introduction

Gum arabic, or gum acacia is mainly derived from Acacia senegal tree. As a nontoxic material it is used as an emulsifier, a thickening agent, and as a stabilizer in foods, with E-code E414 [1]. It is useful in many kinds of foodstuffs because of its very low viscosity, complete solubility in water, and absence of any taste or odour. Due to technical properties gum arabic can be used in multiple applications like in pharmaceutical industry, lithography, and cosmetics. 

Gum arabic is comprised of sugars and glucuronic acid residues in a long chain of galactosyl units with branched oligosaccharides attached to a polypeptide backbone. Protein content of gum arabic varies from 1 to 2%. IgE antibodies against polypeptide chains in gum arabic have been described to elicit asthma in occupational exposure [2]. Occasional cases of occupational asthma in printers [3], candy factory [4, 5], and pharmaceutical industry workers [2] have been described. Although gum arabic is extensively used in food industry ingestion of it is a rare cause of immediate allergy symptoms [1]. 

We describe several cases of occupational asthma caused by gum arabic among candy factory workers.

## 2. Methods

### 2.1. Patients

Eleven candy factory workers with respiratory and/or skin symptoms were referred to the Allergy Unit of Turku University Central Hospital (Table 1).

### 2.2. Workplace Description in the Candy Factory

This Finnish candy factory was a major producer of multiple goodies in Finland since 1910. Gum arabic was an important ingredient in many candies. In making soft pastilles, gum arabic was dissolved in water as kibbles without making dust in the air. Hard boiled candies, instead, were covered with spray dried gum arabic inside a rotating drum. Dry, powdered gum arabic, packed in 25 kg bags, was poured by workers into the drums. Cornstarch, used to cover liquorice, also made dust in the air.

Working clothes were a short-sleeve jacket and/or T-shirt and pants. The workers, who had to do cleaning used rubber gloves. Other protective equipment was not required but respiratory masks were available. 

These patients were referred during the last operation year of the factory. In the preceding year the production of the candy factory had been cut down stepwise before ending of the production. Hard boiled candies were the last products of the factory.

### 2.3. Lung Function Tests

Patients with lower respiratory tract symptoms underwent spirometries and bronchodilation tests with 0.4 mg inhaled salbutamol aerosol administered with a spacer. The dosimetric bronchial histamine challenge test using four stepwise increasing doses of histamine diphosphate solution (0.025, 0.1, 0.4 and 1.6 mg) was carried out using Spiro Electro 2 dosimeter (Spira Respiratory Care Center ltd, Hämeenlinna, Finland) as described by Sovijärvi et al. [6]. The patients were defined to have bronchial hyperresponsiveness if the provocative dose of histamine diphosphate causing a 15% fall in FEV1 (PD15) was 1.6 mg or less. Serial peak expiratory flow measurements (PEF) were carried out in every two hours during the awaketime for a minimum of two weeks period at work and home including at least two periods of free days [7]. PEF record was considered compatible for occupational asthma if there was at least 20% diurnal variation in two working days and less variation in free days and suggestive if there was not over 20% variation but the variation was clearly higher on working days. PEF record was not compatible with occupational asthma if no clear difference were found between working and free days. The fraction of nitric oxide in the exhaled air (eNO) was measured with Niox Mino portable device according to the instructions of the manufacturer.

### 2.4. Specific Bronchial Provocation Test

Specific bronchial provocation tests were performed in the Finnish Institute of Occupational Health in Helsinki with powdered gum arabic and by using lactose powder as a referent test. The provocation tests were done in a 8 m^3^ challenge chamber according to international guidelines [8]. In both the active and the referent tests, the patient sat in the chamber for 30 minutes with the powder bowl in the front of her. The powder was dispersed in the air with compressed air once in every one to five minutes. PEF and FEV1 values were monitored for 24 hours after each challenge test with a pocketsize spirometer (One Flow, Sti Medical, Saint-Romans, France). A >20% fall in FEV1 or PEF values was considered as significant. The patient was also followed for clinical symptoms and lung auscultation.

### 2.5. Skin Prick Tests (SPT)

SPTs were carried out with commercial common environmental allergens including birch, grass and mugwort pollens, cat, dog and horse epithelium, house dust mite, molds and latex (ALK-Abelló A/S, Hosholm, Denmark and concerning birch and timothy from February 2006 to October 2006 Allergopharma, Reinbek, Germany), and with gum arabic (Caesar & Loretz GmbH D-40721) 1 : 10 (w : v) in physiologic saline using a commercial one-peak lancet and prick-prick method. Depending on the exposure in the working place own powdered gum arabic and cornstarch and carmine red colour, all moisturized with saline, were also tested with SPT. Histamine dihydrochloride 10 mg/mL (ALK-Abello) was used as a positive control and the diluent (Soluprick, ALK-Abello) as a negative control. The largest diameter and the diameter opposite to it were measured at 15 min. A reaction was interpreted as positive when the mean of the wheal diameters was at least 3 mm greater than that of the negative control.

### 2.6. Cutaneous Exposure Test

Open cutaneous application test was done on a skin area about 5 cm in diameter on the volar surface of the arm. Gum arabic powder (5 g) moisturized with saline was applied and gently removed at 15 min for the reading of the reaction. In addition to erythema an appearance of one or more wheals was interpreted as a positive reaction.

### 2.7. Patch Tests

Patch testing was carried out to one patient with eczematous skin disease according to standardized guidelines [9]. The allergens were derived from Chemotechnique (Vellinge, Sweden), and the application time was 48 hours. The final interpretation of the test reactions was done at 96 hours.

### 2.8. Serological Tests

Gum arabic specific IgE (Immuno CAP f297, Phadia) was measured in patients with suspected occupational asthma.

### 2.9. Definition of Occupational Asthma

The subject was defined to have occupational asthma due to gum arabic if the asthmatic symptoms worsened at the working place, there was positive skin prick test or specific IgE to gum arabic and a compatible PEF record with occupational asthma and/or positive challenge test. The aim was to confirm all cases by placebo-controlled bronchial challenge tests. One patient was not tested because her PEF recording was compatible with occupational asthma and she had strong oral symptoms of ingested gum arabic.

## 3. Results

### 3.1. Diseases of Candy Factory Workers

Six candy factory workers had occupational allergic disease (Table 1). Four patients had occupational asthma caused by gum arabic. Concomitant occupational contact urticaria was verified by the cutaneous exposure test in 2/4 of them and occupational rhinitis together with asthma in one of them in the specific bronchial provocation test. One other patient had occupational allergic contact dermatitis caused by thiuram chemicals. One patient had occupational urticaria caused by allergy to carmine red used in candies. There were no positive SPT reactions to cornstarch. No work-related allergies were found in five patients. Their symptoms were not clearly related to work and they were not sensitized to work-related allergens. One of them had atopic asthma, one had laryngitis probably caused by reflux disease and smoking and, one had rhinitis which was found not to be related to work. Two patients had chronic urticaria not related to work.

### 3.2. Occupational Asthma due to Gum Arabic in Four Candy Factory Workers

**Figure 1:**
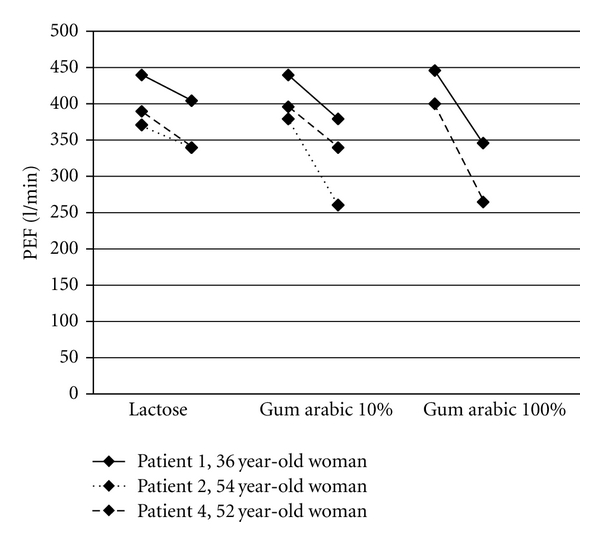
Change in PEF in the challenge tests with lactose (negative control), gum arabic 10%, and gum arabic 100%. All reactions to gum arabic were immediate reactions. The numbers of the patients refer to the numbers in Table 2.

**Figure 2:**
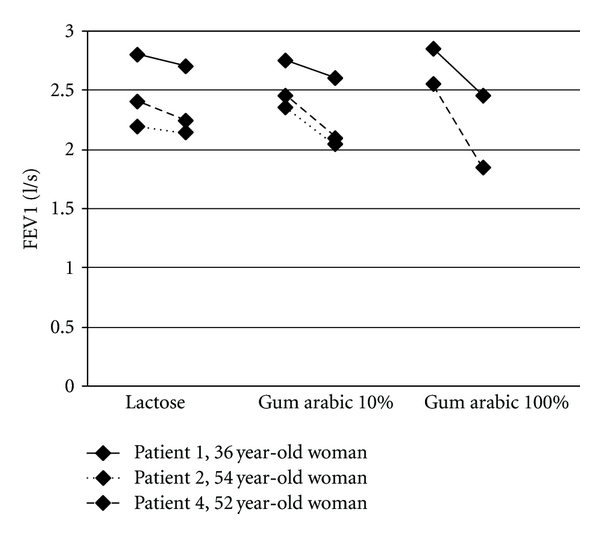
Change in FEV1 in the challenge tests with lactose (negative control), gum arabic 10%, and gum arabic 100%. All reactions to gum arabic were immediate reactions. The numbers of the patients refer to the numbers in Table 2.

The workers with occupational asthma had been doing the same work for 10 to 21 years (mean 14.8 years) and experienced symptoms of the respiratory tract and skin for 1.5 to 10 years (mean 5.1 years). The characteristics of four candy factory workers with occupational asthma due to gum arabic are seen in Table 2.  Figure 1 presents the change in PEF and Figure 2 the change in FEV1 in the challenge tests with lactose (negative control), gum arabic 10%, and gum arabic 100% in patients 1, 2, and 4.

### 3.3. Outcome of the Patients with Occupational Diseases

Patient 1 (in Table 2) with occupational asthma started re-education. The three other patients with occupational asthma continued to work in the factory until the production ended a few months later. They avoided exposure to gum arabic. Six months after the work in the candy factory had ended patient 3 (in Table 2) was free of symptoms without asthma medication. She, however, experienced angioedema when ingesting gum arabic containing foods. In patients 1, 2, and 4 asthma was in control with medication, and they did not experience symptoms associated with gum arabic ingestion.

The patients with occupational skin diseases; caused by carmine red in one and by rubber chemicals in the other; were symptom-free when avoiding those allergens.

## 4. Discussion

Occupational asthma due to IgE-mediated allergy to gum arabic was diagnosed in four candy factory workers. Only one corresponding case has been reported in another Finnish candy factory [4], although the use of gum arabic in food industry is extensive [1]. Even though the occurrence of occupational allergy due to gum arabic is rare, it is possible that there is underreporting of the symptoms. In this study we did not survey the workers in the factory for sensitization to gum arabic and associated symptoms because of the approaching closure of the factory. We do not know whether more workers were sensitized to gum arabic and whether there were mildly symptomatic subjects who had not contacted a doctor. 

Rhinitis is known to increase the risk of asthma by 3 to 5 times [10], and patients with occupational rhinitis have an increased risk of developing asthma [11]. Our patients had also rhinitis and skin symptoms. They had experienced symptoms for a variable, but rather long time before contacting the doctor. They probably did not contact the doctor until they had developed asthmatic symptoms.

All cases of occupational asthma in this factory were caused by gum arabic. In different candy factories carmine, pectin [12], milk, egg, nuts, seeds [13], spices, flavours, guar gum, and cornstarch may cause occupational allergy or nonspecific respiratory symptoms. In this study one worker, sensitized to carmine, was diagnosed to have work associated urticaria with minor respiratory symptoms. The workers who were diagnosed to have occupational asthma due to gum arabic had most symptoms at the working place when handling gum arabic powder. 

SPTs with cornstarch were negative. Airborne cornstarch evidently caused mucosal irritation. We do not know whether exposure to cornstarch powder increased the symptoms due to gum arabic like airborne cornstarch seems to increase symptoms of latex allergy [14]. 

Latex must also be considered in candy factory workers as a cause of occupational urticaria and dermatitis, rhinitis, and asthma. In this study thiuram chemicals in rubber gloves had caused allergic contact dermatitis in one worker but all workers had a negative SPT to latex.

There is an exposure-response relationship between exposure to protein allergens such as wheat flour and alpha-amylase in bakeries and the development of occupational sensitization and symptoms [15, 16]. The production of this candy factory was concentrated on hard pastilles before closing the factory which increased the exposure to gum arabic. Increasing exposure to gum arabic probably caused the symptoms. The workers reported most symptoms in situations where exposure to gum arabic powder was the highest.

The sensitization route of these workers was the respiratory tract and/or the skin. Sensitization through the respiratory tract or skin to a food allergen may lead to the subsequent development of symptoms during oral exposure as has been reported for sensitization to egg in bakery and confectionery workers [17], for lupine seeds in legume laboratory workers [18], for carmine in a worker engaged in dye manufacturing [19] and for fish [20]. Only one of these candy factory workers with occupational asthma reported oral symptoms associated with ingested gum arabic. Despite her asthma relieved after discontinuation of the exposure, the oral symptoms remained.

We have shown that gum arabic may cause occupational allergic rhinitis and asthma with urticaria symptoms. In this study the cases of occupational asthma in the candy factory appeared when there was an increase in the exposure. None of the patients had any previous atopic disease. Working methods which produce less powder and respiratory and skin protection are recommended. Early diagnosis of occupational allergy due to gum arabic is important in order to prevent the development of asthma.

## Figures and Tables

**Table 1:** Patients of the candy factory examined for suspected occupational disease.

Patient sex/age years	Years in candy factory	Duration of symptoms	Symptoms	Diagnosis
F/41	8	6 months	Hives	Chronic nonoccupational urticaria
M/31	10	2.5 years	Hives	Work associated urticaria (carmine and house dust mites positive in skin prick tests)
F/32	8	2 months	Hives	Chronic non-occupational urticaria
F/50	16	9 months	Papular erythema of the hands	Occupational allergic eczema from rubber gloves
F/36	15	1.5 years	Dyspnoea, rhinitis, eye symptoms, hives	Occupational asthma, rhinitis and urticaria from gum arabic
F/54	10	6 years	Dyspnoea, rhinitis, eye symptoms, redness of the skin	Occupational asthma from gum arabic
F/43	13	3 years	Dyspnoea, rhinitis, eye symptoms, itching of the skin	Occupational asthma and urticaria from gum arabic
F/52	21	10 years	Dyspnoea, rhinitis, eye symptoms, hives	Occupational asthma from gum arabic
F/45	Unknown	2 months	Dyspnoea and cough	Laryngitis (non-occupational)
F/35	Unknown	4 months	Dyspnoea and cough	Allergic non-occupational asthma
F/38	10	3 years	Nasal congestion and secretion	Rhinitis (non-occupational)

**Table 2:** The characteristics of four candy factory workers with occupational asthma due to gum arabic.

	Patient 1 36 year-old woman	Patient 2 54 year-old woman	Patient 3 43 year-old woman	Patient 4 52 year old woman
Duration of current work/duration of symptoms (years)	15/1.5	10/6	13/3	21/10
Sensitization to common allergens^1^	No	no	no	no
S-IgE (kU/l)^2^	78	39	104	338
SPT to gum arabic (mm)^3^	4	3	5	3
S-IgE to gum arabic (kU/l)	4.4	0.60	4.5	5.6
FEV1 liters (% of reference )	2.0 (64%)	2.5 (81%)	2.6 (86%)	2.3 (77%)
Compatibility of PEF records in working and free days to occupational asthma^4^	not compatible	suggestive	compatible	compatible
Exhaled NO (ppb)	43	21	20	19
Bronchial hyperresponsiveness in histamine challenge^5^	no	mild	mild	moderate
Bronchial challenge test with gum arabic	positive	positive	not done	positive

^1^Tree, grass and mugwort pollens, cat, dog and horse epithelium, house dust mite, molds, ^2^Serum total immunoglobulin E level, ^3^SPT = skin prick test, ^4^PEF = peak expiratory flow, ^5^strong hyperresponsiveness: histamine PD15 <0.1 mg, moderate hyperresponsiveness: histamine PD15 0.11–0.4 mg, mild hyperresponsiveness: histamine PD15 = 0.41–1.60 mg, no hyperresponsiveness: histamine PD15 >1.60 mg.
